# A Stability Indicating Method for the Determination of the Antioxidant Sodium Bisulfite in Pharmaceutical Formulation by RP-HPLC Technique

**DOI:** 10.3797/scipharm.1104-13

**Published:** 2011-08-07

**Authors:** Harshal Kanubhai Trivedi, Mukesh C. Patel

**Affiliations:** 1 Analytical Research Lab, Cadila Pharmaceutical Ltd, Dholka-387 810, Gujarat, India; 2 P.S. Science and H.D. Patel Arts College, S.V. Campus, Kadi-382 715, Gujarat, India

**Keywords:** Inorganic compound, Chromatography, Antioxidant, Assay, Method validation, Amikacin sulfate injection

## Abstract

A stability-indicating reversed-phase high-performance liquid chromatographic (RP-HPLC) method was developed for the determination of sodium bisulfate (SB), an antioxidant, in injectable dosage form. The chromatographic separation was achieved on a Zorbax CN (250 mm × 4.6 mm, 5 μm) column, with a mobile phase consisting of a buffer mixture of 0.03 M tetrabutylammonium hydrogen sulfate, 0.01 M potassium dihydrogen orthophosphate, and acetonitrile at a ratio of 70:30 (v/v) and a flow rate of 0.7 mL/min. The eluted compound was monitored at a wavelength of 215 nm using a UV detector. The method described herein separated sodium bisulfite from all other formulation components within a run time of 10 min. The method also generated linear results over an SB concentration range of 10 to 990 μg/mL, and the limit of quantification was found to be 10 μg/mL. The stability indicating capability of the method was established by performing forced degradation experiments. The RP-HPLC method that was developed was validated according to the International Conference on Harmonization (ICH) guidelines. This method was successfully applied in the quantitative determination of SB in a stability study of Amikacin sulfate injection. The procedure described herein is simple, selective, and reliable for routine quality control analysis as well as stability testing.

## Introduction

**Fig. 1:**
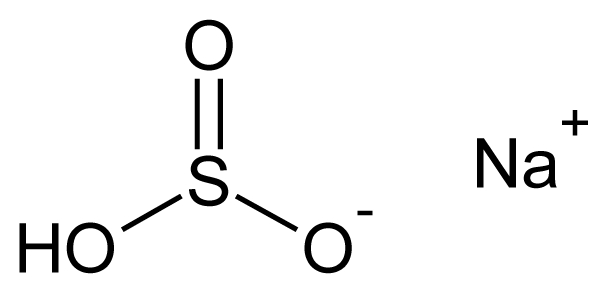
Chemical structure of sodium bisulfite

Sodium bisulfite (SB) is an inorganic compound commonly used as an antioxidant in pharmaceutical formulations. Antioxidants are excipients that are used to improve the stability of medicines by delaying the oxidation of active substances and other excipients and are classified into 3 groups [1]. The first group is known as true antioxidants, or anti-oxygen, which likely inhibit oxidation by reacting with free radicals and blocking chain reactions. The second group consists of reducing agents; these substances have lower redox potentials than the drug or adjuvant that they are intended to protect and are therefore more readily oxidized. Reducing agents may also operate by reacting with free radicals. The third group consists of antioxidant synergists that usually have little antioxidant effect themselves but are thought to enhance the action of antioxidants in the first group by reacting with heavy metal ions that catalyze oxidation. The chemical structure of SB is presented in Figure 1.

SB is a common reducing agent that is used in both chemical and pharmaceutical industries. It readily reacts with dissolved oxygen and is converted into sodium hydrogen sulfate [2].

$$2\;{\text{NaHSO}}_{3}+{\text{O}}_{2}\to 2\;{\text{NaHSO}}_{4}$$

SB is oftentimes added to large piping systems to prevent oxidative corrosion. In biochemical engineering applications, SB is helpful in maintaining anaerobic conditions within a reactor. The antioxidant properties are due to certain chemical groups which are usually harmful to living cells and might therefore be associated with certain risks when used in humans [3]. Thus, inclusion of antioxidants in any finished products needs special justification. Finished product release specifications should include an identification test and a content determination test with acceptance criteria and limits for each antioxidant present in a formulation. The finished product’s shelf-life specification should also include an identification test and limits for any antioxidants present [3]. Whenever antioxidants are expended during the manufacturing of a product, the release limits should be justified by batch data. The adequacy of specified limits should be justified on the basis of controlled conditions and in-use stability testing to ensure that sufficient antioxidant remains to protect the product throughout its entire shelf-life and during the proposed in-use period [3]. The antioxidant properties of SB are therefore an integral part of product formulation. This concept encourages the development of new a stability-indicating method for the estimation of SB in today’s chemical and pharmaceutical industries.

A detailed literature survey for SB revealed that a spectroscopic method is available for the determination of sulfite content in aqueous medium using Ellman’s reagent [4]. Another method allows for the determination of sodium metabisulfite in parenteral formulations by high performance ion chromatography [5]. In this technique, the correlation coefficient was found to be less (>0.99), the sample precision value was higher than 6.0 % and the stability-indicating capability of the method was not demonstrated according to ICH guidelines [6]. Moreover, SB is not officially represented in any pharmacopoeia to date. There is no stability-indicating HPLC method reported in the literature that can adequately separate and accurately quantify SB in Amikacin injection, thus necessitating the development of a new stability-indicating method to assay SB in pharmaceutical formulation.

The purpose of this study was to develop a stability-indicating method for the determination of SB in injection formulation. The method developed was able to separate SB from Amikacin sulfate and other excipients of a drug product within 10 min. Upon successful separation, this technique was validated as per ICH guidelines [6] and successfully applied in the separation and quantification of SB in Amikacin sulfate injections.

## Results and Discussion

### Method development and optimization

The main criterion for developing an RP-HPLC method for the determination of SB as an inorganic compound using a UV detector was to estimate the amount of SB in a single run, with emphasis on the method being accurate, reproducible, robust, stability indicating, linear, free of interference from other formulation excipients and convenient enough for routine use in quality control laboratories.

**Fig. 2:**
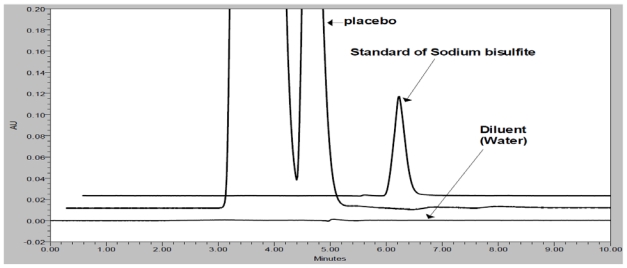
Overlaid chromatograms of placebo, blank and standard (SB)

**Fig. 3:**
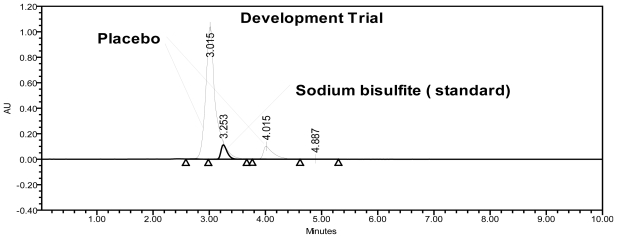
Overlaid chromatograms (unsuccessful conditions) of standard (SB) and sample solution.

A spiked solution of SB (660 μg/mL) and placebo peaks were subjected to separation by RP-HPLC. Initially, the separation of all peaks was studied using water as mobile phase A and acetonitrile as mobile phase B on an HPLC column (Hypersil BDS-C18) and Waters (HPLC) system with a linear gradient program. The 0.5 mL/min flow rate was selected to achieve the separation of peaks. The column oven temperature was maintained at 25°C. These conditions resulted in merging of the SB peak with the placebo peaks, represented in Figure 3. Based on this result, the C18 column was replaced with a polar cyano column in an effort to achieve high resolution between the placebo peak and the SB peak. With the cyano column (Zorbax CN), different combinations of mobile phase A and B were studied to optimize the method, and the results of the optimization are summarized in Table 2, including any observations noted. From the mobile phase selection study, the optimized HPLC parameters were as follows: flow rate, 0.7 mL/min; column oven temperature, 30°C; injection volume, 10 μL; and an isocratic program with a mixture of buffers (0.03 M tetrabutylammonium hydrogen sulfate (TBAS) and 0.01 M potassium dihydrogen orthophosphate in Milli-Q water adjusted to pH 6.0 with orthophosphoric acid) and acetonitrile in the ratio of 70:30 (v/v) as the mobile phase. The column oven temperature was also studied; it was found that 30°C was a more appropriate temperature with respect to peak separation and shape. Based on the UV spectrum of the compound, 215 nm was found to be appropriate for the determination of SB in pharmaceutical formulations. SB and other excipients are well resolved with respect to each other in a reasonable time of 10 minutes (Figure 2). No chromatographic interference due to the blank (diluent) and other excipients (placebo) at the retention time of SB was observed, as shown in Figure 2.

### Analytical parameters and validation

After development, this method was subjected to validation according to ICH guidelines [6]. The method was validated to demonstrate that it is suitable for its intended purpose by the standard procedure to evaluate adequate validation characteristics (system suitability, accuracy, precision, linearity, robustness, solution stability and stability-indicating capability).

### System suitability

The percentage relative standard deviation (RSD) of area from six replicate injections was below 2.0 %. Low values of RSD for replicate injections indicate that the system is precise. The results of other system suitability parameters such as peak tailing and theoretical plates are presented in Table 3. As seen from this data, the acceptable system suitability parameters would be as follows: the relative standard deviation of replicate injections is not more than 2.0 %, the tailing factor for the peak of SB is not more than 1.5 and the theoretical plates are not less than 2000.

### Specificity

**Fig. 4:**
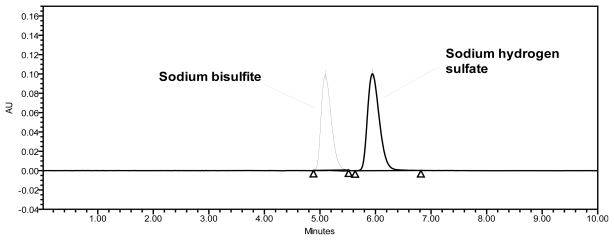
Overlaid chromatograms of SB and sodium hydrogen sulfate

Forced degradation studies were performed to demonstrate the selectivity and stability-indicating capability of the proposed RP-HPLC method. Figure 2 shows that there is no interference at the RT (retention time) of SB from the blank and other excipients. Significant degradation was not observed when SB was subjected to acid, base, thermal, hydrolytic and UV conditions, whereas significant degradation was observed when the SB was subjected to oxidative hydrolysis (3% H_2_O_2_, 60°C, 30 minutes), leading to the formation of sodium hydrogen sulfate. The oxidative product (sodium hydrogen sulfate) and SB are well separated from each other, as seen in Figure 4. The peak attributed to SB was investigated for spectral purity in the chromatogram of all exposed samples and was found to be spectrally pure. The purity and assay of SB was unaffected by the presence of other excipients and thus confirms the stability-indicating power of this method. The results of the forced degradation study are presented in Table 4.

### Limit of quantification (LOQ)

The concentration (in μg/mL) with a signal to noise ratio (S/N) of at least 10 was taken as the LOQ, which meets the criteria defined by ICH guidelines. The LOQ for the SB peak was found to be 10 μg/mL. The precision was also established at the quantification level. The % RSD of the peak area was well within the acceptance limit of <10.0 %. The determined limit of qualification and precision at LOQ values for SB is presented in Table 5.

### Linearity

**Fig. 5:**
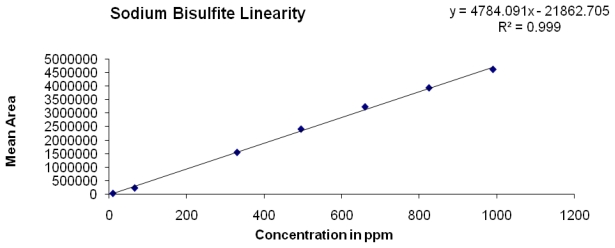
Linearity of SB

The linearity of an analytical method is its ability to elicit test results that are directly, or by a well-defined mathematical transformation, proportional to the concentration of analyte in that sample within a given range. The response was found to be linear from 1.5 % to 150 % of standard concentration. The regression statistics are shown in Table 6, with the linearity curve for SB represented in Figure 5.

### Precision

The purpose of this study was to demonstrate the reliability of the test results with variations. The average % assay (n = 6) of SB was 99.8 % with RSD of 1.0 %. The results are shown in Table 7, along with intermediate precision data. Low RSD values indicate that this method is precise.

### Accuracy

The accuracy of an analytical method is the closeness of test results obtained by that method compared with the true values. The amount recovered (for 10, 50, 100 and 150 % level) was within ± 2 % of amount added; for the LOQ level, the amount recovered was within ± 10 % of the amount added, indicating that the method is accurate and that there is no interference due to other excipients present in the injection. The results of the recovery assay are shown in Table 8.

### Robustness

The robustness of an analytical procedure is a measure of its capacity to remain unaffected by small but deliberate variations in method parameters and provides an indication of its reliability during normal usage. No significant effect was observed on system suitability parameters such as RSD, tailing factor, or the theoretical plates of SB when small but deliberate changes were made to chromatographic conditions. The results are presented in Table 3, along with the system suitability parameters of normal conditions. Thus, the method was found to be robust with respect to variability in applied conditions.

### Stability of the sample solution

Drug stability in pharmaceutical formulations is a function of storage conditions and chemical properties of the SB. Conditions used in stability experiments should reflect situations likely to be encountered during actual sample handling and analysis. Stability data are required to show that the concentration and purity of analyte in the sample at the time of analysis corresponds to the concentration and purity of analyte at the time of sampling. A sample solution did not show any appreciable change in assay value when stored at ambient temperature up to 24 h (Table 9). The results from solution stability experiments confirmed that the sample solution was stable for up to 24 h during the assay procedure.

### Application of the method to stability study

The present method was applied for the estimation of SB during a stability study. The results obtained are presented in Table 10.

## Experimental

### Materials and Reagents

Amikacin sulfate injection and placebo solution were provided by Cadila Pharmaceutical Ltd. Dholka, Ahmedabad, India, along with the working standard. HPLC grade acetonitrile and methanol were obtained from J.T. Baker (NJ., USA). GR grade potassium dihydrogen phosphate, tetrabutylammonium hydrogen sulfate and orthophosphoric acid were obtained from Merck Ltd. (Mumbai, India). Nylon membrane filters (0.22 μm) and nylon syringe filters were purchased from Pall Life Science Limited (India). High purity water was generated with Milli-Q Plus water purification system (Millipore, Milford, MA, USA).

### Buffer preparation

A solution of 0.01 M phosphate buffer (KH_2_PO_4_) and 0.03 M tetrabutylammonium hydrogen sulfate was prepared using Milli-Q water. The pH was adjusted to 6.0 with orthophosphoric acid. The buffer preparation was stable with respect to pH and maintained visual clarity for 48 h.

### Chromatographic conditions

Analysis was performed on an Alliance Waters HPLC system consisting of a quaternary solvent manager, sample manager, and PDA (photo diode array) detector. System control, data collection, and data processing were accomplished using Waters Empower chromatography data software. The chromatographic conditions were optimized on an Agilent Zorbax CN (250 mm × 4.6 mm, 5 μm) column. The mobile phase was a mixture of buffer and acetonitrile at a ratio of 70:30 (v/v). The mobile phase was filtered through 0.22 μm nylon membrane filter and degassed under vacuum prior to use. Purified water was used as a diluent. The optimized conditions were as follows: an injection volume of 10 μL, isocratic elution at a flow rate of 0.7 mL/min, 30°C (column oven) temperature, and 215 nm detection wavelength. Under these conditions, the backpressure in the system was approximately 2,000 psi. The stress degraded samples were analyzed using a PDA detector over a range of 200–400 nm.

### Standard solution preparation

The standard solution was prepared by dissolving the standard in diluent to obtain a solution containing 660 μg/mL of SB.

### Sample solution preparation

For the preparation, 2.0 mL of sample solution was accurately transferred into a 20 mL volumetric flask. Approximately 15 mL of diluent was added to the volumetric flask, which was then sonicated in an ultrasonic bath for 3 min. The resulting solution was then diluted up to the mark with diluent and mixed well.

### Placebo solution preparation

In preparing the placebo solution, 2.0 mL of placebo solution was accurately transferred into a 20 mL volumetric flask. Approximately 15 mL of diluent was added to the volumetric flask, which was then sonicated in an ultrasonic bath for 3 min. The resulting solution was then diluted up to the mark with diluent and mixed well.

### Method validation

The method described herein has been validated for assay determination by HPLC.

### System suitability

System suitability parameters were performed to verify the system performance. System precision was determined on six replicate injections of standard preparations. All the important characteristics, including the relative standard deviation, peak tailing, and theoretical plate number, were measured.

### Specificity

Forced degradation studies were performed to demonstrate selectivity and stability-indicating the capability of the proposed method. The sample was exposed to acidic (0.5 N HCl, 60 °C, 1 h), alkaline (0.5 N NaOH, 60 °C, 1 h), strong oxidizing (3 % H_2_O_2_, 60 °C, 30 min), thermal (60 °C, 6 h) and photolytic (UV) degradation conditions. All exposed samples and standards were then analyzed by the proposed method.

### Limit of quantification (LOQ)

The LOQ was determined using a signal to noise approach as defined in the International Conference on Harmonization (ICH) guidelines [6]. A serially diluted solution of SB was injected into the chromatograph and the signal to noise (S/N) ratio was calculated at each concentration.

### Linearity

Linearity was demonstrated from 1.5 to 150 % of standard concentration using a minimum of seven calibration levels (1.5 %, 10 %, 50 %, 75 %, 100 %, 125 % and 150 %) for SB. The method of linear regression was used for data evaluation. The peak area of the standard compound was plotted against the respective SB concentrations. Linearity was described by the linearity equation and the correlation coefficient was also determined.

### Precision

The precision of the system was determined using the sample preparation procedure described above for six real samples of Amikacin sulfate injection and analysis using the same proposed method. Intermediate precision was studied using different columns and was performed on different days.

### Accuracy

To confirm the accuracy of the proposed method, recovery experiments were carried out by the standard addition technique. Five levels (LOQ, 10 %, 50 %, 100 % and 150 %) of standards were added to pre-analyzed samples in triplicate. The percentage recoveries of SB at each level and each replicate were determined. The mean of percentage recoveries (n = 15) and the relative standard deviation were also calculated.

### Robustness

The robustness is a measure of the capacity of a method to remain unaffected by small but deliberate changes in flow rate (± 0.1 mL/min), change in column oven temperature (± 5 °C), change in pH of buffer (± 0.1) and change in wavelength nm (± 2 nm).

### Stability of sample preparation

The stability of the sample solution was established by storage of the sample solution at ambient temperature for 24 h. The sample solution was re-analyzed after 24 h, and the results of the analysis were compared with the results of the fresh sample.

## Conclusion

A new RP-HPLC method was successfully developed for the estimation of sodium bisulfite in Amikacin Injection. The method validation results have verified that the method is selective, precise, accurate, linear, robust and stability indicating. The run time (10.0 min) enables rapid determination of SB. This stability-indicating method can be applied for the determination of sodium bisulfite in release testing and in stability studies of Amikacin Injection. Moreover, it may be applied for the determination of SB in bulk drugs, chemical processing or in reverse engineering techniques to identify reacted and un-reacted quantities of SB.

## Figures and Tables

**Tab. 1:** Label claim of SB with its working concentration (specification limit)

Compound	Label claim mg/mL	Working concentration	
		(mg/mL)	(μg/mL)
SB	6.6 mg	0.66	660

**Tab. 2:** Summary of method optimization

Experimental condition	Observation
Water(MP-A) and acetonitrile (MP-B), linear gradient; Hypersil BDS-C18 (250 mm × 4.6 mm, 5 μm); 25°C	SB peak was merged with placebo peak
0.01 M KH_2_PO_4_ (MP-A) and acetonitrile (MP-B), linear gradient; Hypersil BDS-C18 (250 mm × 4.6 mm, 5 μm); 25°C	SB peak was merged with placebo peak
0.01 M KH_2_PO_4_ + 0.03 M TBAS (MP-A) and acetonitrile (MP-B), linear gradient; Hypersil BDS- C18 (250 mm × 4.6 mm, 5 μm); 25°C	Slight peak separation was observed
0.01 M KH_2_PO_4_ + 0.03 M TBAS (MP-A) and acetonitrile (MP-B), linear gradient; Zorbax CN (250 mm × 4.6 mm, 5 μm); 25°C	SB peak was separated from placebo
0.01 M KH_2_PO_4_ + 0.03 M TBAS, pH 6.0 with H_3_PO_4_ (buffer) and acetonitrile, (70:30) v/v; Zorbax CN (250 mm × 4.6 mm, 5 μm); 30°C	Satisfactory peak separation and peak shape

**Tab. 3:** System suitability results (precision, intermediate precision and robustness)

Parameter	Theoretical plates*	Tailing factor*	% RSD* of standard
Precision	2950	1.1	1.00
Intermediate Precision	3222	1.0	0.72
At 0.6 mL/min flow rate	2604	1.2	0.54
At 0.8 mL/min flow rate	2734	1.1	0.82
At 25°C column temp.	2678	1.1	0.67
At 35°C column temp.	2879	1.0	0.78
At buffer pH 5.9	2750	1.1	1.10
At buffer pH 6.1	2938	1.1	0.71
At 213 nm	2812	1.2	0.53
At 217 nm	2910	1.2	0.58

* Determined on six values.

**Tab. 4:** Summary of forced degradation results

Degradation condition	Assay (% w/w)	Purity Flag	Observation
Control sample	99.8	No	Not applicable
Acid hydrolysis (0.5 N HCl, 60°C, 1 h)	98.5	No	SB found stable
Alkaline hydrolysis (0.5 N NaOH, 60°C, 1 h)	98.4	No	SB found stable
Oxidation (3 % H_2_O_2_, 60°C, 30 min)	78.3	No	SB found sensitive
Thermal (60 °C, 6 h)	100.8	No	SB found stable
Exposed to UV at 254 nm	100.1	No	SB found stable

**Tab. 5:** LOQ and its precision results

Substance	LOQ (μg/mL)	S/N	Precision (% RSD*)
SB	10	10.2	2.3 %

* Determined on six values

**Tab. 6:** Regression statistics

Subst.	Linearity range (μg/mL)	Correlation Coefficient (R^2^)	Linearity (Equation)	Y-intercept bias in %	p-value*
SB	10 to 990	0.999	y = 4784.091X – 21862.705	0.7	0.0001

* Calculated by Statistical Analytical Software, Version 9.2

**Tab. 7:** Precision (660 μg/mL) and Intermediate precision (660 μg/mL) results

Substance	Precision		Intermediate precision	

	% Assay #	% RSD*	% Assay #	% RSD*
SB	99.8	1.0	101.0	0.7

# Average of six determinations;

* Determined on six values.

**Tab. 8:** Accuracy results of SB

	At LOQ 10 μg/mL	At 10 % 66 μg/mL	At 50 % 330 μg/mL	At 100 % 660 μg/mL	At 150 % 990 μg/mL
	
% Recovery#	103.6	101.6	99.7	98.5	98.6
% RSD*	3.0	0.44	0.91	0.85	0.60

* Determined on three values;

# Mean of three determinations.

**Tab. 9:** Solution stability results

**% Assay**	**Initial**	**After 24 hrs.**

	101.2	100.9

**Tab. 10:** Results of stability study (Amikacin injection)

Sample ID	% Assay of SB
Initial	99.8 %
1 M 40 °C /75 % RH	85.3 %
2 M 40 °C /75 % RH	77.2 %
3 M 40 °C /75 % RH	62.5 %
6 M 40 °C /75 % RH	46.3 %
